# Subscription Bills and an Explanation

**Published:** 1896-11

**Authors:** 


### SUBSCRIPTION BILLS AND AN EXPLANATION.

Upon the back of every bill sent to subscribers in October appeared a polite request for settlement and a statement as to choice of those who were very far behind settling with us or a collection agency. Some few have gotten offended at the latter without just cause. We could not state on the bill how much it took to make one "very much behind." "Very much behind" probably did not apply to over twenty-five men. A bill is never turned over to a collecting agency until its payment seems hopeless, or until the account has run some few years.

Another thing: THE JOURNAL is run with absolute honesty as far as we know it. If a subscriber feels that his account is unjust or he is overcharged, we always give him the benefit of any doubt. On many pages of our books appear such memoranda as these: "Allow claim to," "Cr. by lost copies," and several stating that "So-and-so is unfortunate or broken down in health, *send him no bill.*" When, through error in our bill, a subscriber overpays his account, we send back the amount overpaid. When a man tells us he moved from the address we had long ago, we frequently credit him with lost copies, even if he has failed to notify us of his change of address.

In short, the case is just this: We don't want a cent over our just dues, we always give the subscriber the advantage and we do not expect ever to do less than what is right.

There are excellent gentlemen who have come, or written, and asked an explanation, have been satisfied and have remained our friends.

Others have acted as if they preferred the privilege of resenting a supposed wrong, stopped their JOURNAL and would accept no explanation. To such as these we can only explain and let them depart in peace. MANAGER.

